# Dimethoate Induced Behavioural Changes in Juveniles of* Cyprinus carpio* var.* communis* under Temperate Conditions of Kashmir, India

**DOI:** 10.1155/2016/4726126

**Published:** 2016-08-03

**Authors:** Imtiyaz Qayoom, Feroz A. Shah, Malik Mukhtar, Masood H. Balkhi, Farooz A. Bhat, Bilal A. Bhat

**Affiliations:** ^1^Faculty of Fisheries, Sher e Kashmir University of Agricultural Sciences and Technology of Kashmir, Rangil, Ganderbal 191201, India; ^2^Research Centre for Residues and Quality Analysis, Sher e Kashmir University of Agricultural Sciences and Technology of Kashmir, Shalimar 190025, India

## Abstract

The present study was designed to investigate acute toxicity of dimethoate on juvenile* Cyprinus carpio *var.* communis*. Fishes weighing 10 ± 2 gms were selected and mortality data was statistically evaluated by Finney's Probit Method. The 96-hour LC_50_ value for* Cyprinus carpio *was found as 1.1 ppm in static bioassay system. Mean values of physicochemical parameters of aquarium waters determined during bioassay depicted slight variation indicating that the mortality in aquarium fishes occurred due to pesticide exposure and not suffocation. Lab. temperature ranged from 12 to 13°C; water temperature ranged from 11 to 12°C; dissolved oxygen ranged from 3.90 to 4.56 mg/L; pH ranged from 6.90 to 7.05; total dissolved solids ranged from 2.66 to 3.0 × 10^3^ mg/L, while CO_2_ remained at a constant value of 2.0 mg/L. The fishes elicited various behavioural responses such as uncoordinated movements, convulsions, excessive mucus secretion, and imbalanced swimming which ended in a collapse to the bottom of the aquarium. Prior to death, the clinical signs like scale erosion, pale body colour, and hemorrhagic patches over the body were noticed which became more vivid up to the termination of experiments. Results of the study indicate potential toxicity of dimethoate in fingerlings of common carp for which the natural waterbodies must be continuously monitored to reduce its impact across food chains.

## 1. Introduction

Jammu and Kashmir is an agricultural state with varied agroclimatic conditions suitable for the cultivation of different types of crops. Kashmir province of the state is well recognized by horticulture, where apple cultivation finds the primary importance. The fruit production of Kashmir province alone is 1.5 million metric tons annually from a total orchard area of 0.2 million hectares, which is sprayed and fogged with 7750 metric tons of fungicides and 3186 metric tons of insecticides, right from March to November every year [1]. In 2009, the total sale of pesticides by weight was 1828.5 thousand kg or liters costing about 369.1 million rupees [2] and is increasing every year. However, the use of pesticides leaves a negative impact on the nontarget species, especially in the aquatic environments. Nonjudicious and unavoidable entrance of pesticides into natural waterbodies through leaching, percolation, precipitation, drift, or runoff leads not only to contamination of natural water resources but also to toxicity to aquatic organisms. Fishes are generally exposed to pesticides through dermal uptake, direct absorption through the skin, or direct uptake through the gills during respiration [1, 3]. Once uptaken through any of the routes, these xenobiotics are potent to cause physiological dysfunctions like oxidative stress [4–6] and haematological [7–10] and biochemical [9, 11] changes in fishes.

Biological assays (or bioassays) are a set of techniques relevant to the comparisons between the strengths of alternative but similar biological stimuli (a pesticide, a fungicide, a drug, a vitamin, plant extract, etc.) based on the response produced by them on the subjects (e.g., an animal, a piece of animal tissue, a plant, a bacterial culture, subhuman primates or humans, living tissues, plants or isolated organisms, insects, etc.). Typically, a bioassay involves stimulus, subject, and a response elicited by the subject due to application of stimulus. It is the estimation of the potency of an active principle in a unit quantity of preparation or detection and measurement of the concentration of the substance in a preparation using biological method. Fish bioassays have been widely used to determine the lethal concentrations of various xenobiotics, their effects on fish physiology, and the level of penetration of these compounds in muscle tissues of fishes. The tests are helpful in estimation of pollution levels in natural waterbodies and residual effects of these chemicals on fishes.

Dimethoate (O,O-dimethyl-S-[2-(methylamino)-2-oxoethyl] phosphorodithioate) is a broad range organophosphorus insecticide described by [12] for the first time. It was introduced in 1956 and is produced in many countries for use against a broad range of insects in agriculture and also for the control of the housefly and other household insects. Its mode of action is acetylcholinesterase (AChE) inhibition resulting in nerve exhaustion, nervous system failure, and ultimately death. Dimethoate is found to exhibit toxicity to terrestrial and aquatic organisms, particularly fishes. It is one of the important organophosphorus insecticides widely used in Kashmir valley sprayed on wide variety of crops, fruits, and vegetables and is rated as one of the most abundantly used organophosphate compounds in Kashmir valley [13]. In this study, acute toxicity of dimethoate on fingerlings of* Cyprinus carpio* var.* communis* was assessed in order to determine the median lethal concentration of the pesticide and the behavioural responses elicited by the fishes.

## 2. Material and Methods

Experiment was carried out as per the method described by [14]. A total number of 360 specimens of* Cyprinus carpio* var.* communis* were brought from hatchery of Faculty of Fisheries, SKUAST-K, to the laboratory in plastic bags with adequate water, avoiding any physical injury to them. The test organisms having weight of 10 ± 2 g were taken only. Care was taken that the length of the largest fish was not more than 1.5 times the length of the smallest fish. Fish samples were disinfected by giving bath in 0.05% KMnO_4_ solution for two minutes to avoid any infection. Prior to the introduction of fishes, aquaria were also washed with KMnO_4_ to avoid infection. Fishes were acclimatized to laboratory environment for two weeks of aquaria with the dimensions of 60 × 30 × 40 cm and fed with artificial diet during that period. The artificial fish feed was brought from Manasbal National Carp Farm Kashmir and comprised of wheat bran (25%), rice bran (25%), crushed maize (20%), mustard oil cake (20%), soya bean meal (6%), linseed oil (35%), and vitamins and minerals (1%) fed once a day at 3% body weight. The leftover matter, whether feed or fecal matter, was thoroughly cleaned and water of the tank was changed in the subsequent morning. The specimens were divided into 2 groups, 180 specimens in each group, used in range finding and definitive tests. Maximum loading of the test organism was 1 g/L and 10 fishes were recruited in each aquarium for range finding test.

### 2.1. Dilution Water

Double distilled water was used as dilution water. Various water quality parameters of aquarium water like temperature, pH, CO_2_, dissolved oxygen, and total dissolved solids were checked every day as per the methodology of American Public Health Association [2, 15]. Temperature of laboratory was also recorded during each bioassay. Addition of the toxicant into the dilution water was followed by swirling of the water with glass rod to disperse the toxicant immediately and uniformly throughout the aquarium. Maximum loading of the test organism was 1 g/L and 10 fishes were recruited in each aquarium to ensure their free movement and avoid any stress due to overcrowding or suffocation. Before the start of experiment, the dilution water was aerated vigorously with glass rod so that the dissolved oxygen should be not less than 6 mg/L at 26°C.

### 2.2. Test Concentration

Technical grade of dimethoate was procured from Hyderabad Chemicals Private Ltd., India, with 90% purity. Stock solutions of dimethoate were prepared in methanol and subsequent concentrations in deionised water. Range finding test was carried out to determine the final concentrations to be used in definitive test. For range finding test of dimethoate, five concentrations were selected based on the literature survey. 0.01, 0.1, 1.0, 10.0, and 100 ppm concentrations were administered in replicates and corresponding mortality was recorded. Finally, for the definitive test, 0.5, 1.0, 2.0, 4.0, and 8.0 ppm concentrations were selected and administered in triplicate, on the basis of which mean lethal concentration of the pesticide was determined. To determine range finding test, concentrations with spacing factor of 10 were used [16].

### 2.3. Bioassay

The bioassay was carried out with the standard ethical protocol of US-EPA EPA-660/3-75-009. Static type of bioassay for 96 hours was carried out. Feeding was stopped 24 hours before the start of experiment. During the experiment also, no food was given to fishes. Test organisms were introduced to the test chamber (aquaria) within one hour after the toxicant was added to the dilution water. Mortality counts were recorded in each concentration at 6, 12, 24, 48, 72, and 96 hours. The five concentrations per toxicant in definitive test and each concentration were replicated thrice with 10 specimens per replicate. Control group was also run in both range finding and definitive tests in which same amount of solvent was added as to the treated one. Addition of solvent to the control was to rule out the mortality caused, if any, due to its toxic effects in the fishes. Fishes were treated dead if any sign of immobilization, loss of equilibrium, lack of opercular movement, or morbidity was seen. This reflected an indication of pending death. Behavioural responses like excess mucus secretion and change in swimming pattern, loss of scales, and change in colour patterns were also taken into consideration and keenly monitored during the entire bioassay. Dead test organisms were removed from aquaria as soon as observed. After the experiment is over, test solution was disposed off and container was scrubbed and washed thoroughly with 10% HCl.

### 2.4. Water Quality Parameters of Aquarium Waters during Bioassay

Various water parameters of aquarium waters were calculated every day during bioassays. Laboratory temperature and water temperature were recorded by using a mercury filled thermometer and results were expressed as °C. pH of the water was determined with the help of pH meter. Free carbon dioxide was measured by using phenolphthalein indicator and sodium hydroxide titrant. Dissolved oxygen was determined by modified Winkler's method and total dissolved solids were determined as per the method described by [2].

### 2.5. Statistical Analysis

Percent mortality data of fish at the end of 96 h exposure were determined by Probit method of regression [17] manually and then confirmed by using R statistical software (3.1 version). For descriptive statistics, that is, calculation of mean and standard deviations, SPSS (20.0 version) was used.

## 3. Results

### 3.1. Median Lethal Concentration (LC_50_) of Dimethoate

The median lethal concentration (LC_50_) values in the present study were determined on the basis of range finding tests done thrice as replicates (*r*_1_, *r*_2_, and *r*_3_) (Table 1). The mean median lethal concentration (LC_50_) value of dimethoate after acute exposure to common carp was 1.1 ppm. The median lethal concentration (LC_50_) values obtained from range finding tests are given in Table 1. A strong correlation between the log dose and probit mortality of fishes was observed.

### 3.2. Water Quality of Aquarium Waters during Dimethoate Bioassay

Various water quality parameters assessed during static bioassay of dimethoate showed a slight variation up to the completion of bioassay. Laboratory temperature ranged from 12 to 13°C, while water temperature varied between 11 and 12°C as the experiment was performed in the month of November. Values of carbon dioxide remained constant throughout the experiment signifying nonhypoxic conditions in the aquarium throughout the assay. Various water quality features of aquarium waters are presented in Table 2.

## 4. Behavioural Responses in Fishes Exposed to Pesticides

The behavioural responses elicited by juveniles of common carp during acute exposure to dimethoate are presented in Table 3. During the static bioassay of dimethoate to* Cyprinus carpio* var.* communis* various behavioural responses were elicited by the fishes. During first 6 hours fishes were seen slowing down their motion which grew more with the advent of time. Uncoordinated movements were seen clearly during first six hours of the experiment in the fishes which later on died. Those fishes which survived during entire bioassay did not show the signs of uncoordinated movements. Convulsions were seen to get started from 12th hour and lasted until the 24th hour. Strong convulsive fits were seen up to fishes elicited at 24th hour of the experiment. The slight secretion of mucus could be seen from the 24th hour which grew more intense with the termination of experiment. Imbalance in the swimming started just within six hours of the start of the experiment which grew intense with erratic and spiral swimming movements and collapse to the bottom of the aquarium ultimately. Prior to death, fishes became inactive and almost nonmotile with clinical signs of erosion of scales and turning of body colour into pale and lesions and hemorrhagic patches all over the body especially on the ventral side.

## 5. Discussion

During present study, the acute toxicity tests were carried out in* Cyprinus carpio* var.* communis* exposed to dimethoate. The median lethal concentration (LC_50_ value) for dimethoate was found to be 1.1 mg/L which is in accordance with the earlier researches by various researchers. The 96 h LC_50_ value of 4.57 mg/L has been reported for* Saccobranchus fossilis* exposed to dimethoate [18], while 0.14 mg/L for* Pteronarcys californica* was observed at 48 hours (48 h LC_50_) [19]. Classifying it as an intermediary toxicity chemical, [20] reported the median lethal concentration of 60.00 *μ*g/L for adults of* Danio rerio*. They also reported LC_50-96-h_ values of 24.64 and 21.64 *μ*g/L for embryo and fingerlings of the fish exposed to the pesticide. The 96 h LC_50_ value of dimethoate in* Clarias batrachus* has been reported as 65 mg/L [21] which is higher than the values obtained in present study and can be attributed to the fishes of large weight used in their study. In* Heteropneustes* 96 h LC_50_ value of dimethoate was found to be 2.98 mg/L [22], while [23] reported 15.92 mg/L for 24 h, 13.42 mg/L for 48 h, 12.39 mg/L for 72 h, and 11.34 mg/L for 96 h in the fish indicating that fishes are much susceptible to the dimethoate toxicity. Reference [24] reported LC_50_ value of dimethoate in 3-day-old* Prochilodus lineatus* larva to be 10.44 *μ*g/L. References [25, 26] estimated LC_50_ value of dimethoate in* Puntius ticto* as 5.012 ppm. The small values of LC_50_ obtained during present study are attributed to small size of fishes (10 ± 2 g) which have potentially weak immune system to carry out the elimination of the toxicant from the body. Moreover, the rapid distribution of pesticides in the body of small sized fishes leads to induction of behavioural changes faster than the normal, since the uptake of a toxicant is directly dependent on the size of fishes.

Water quality parameters of aquarium during present study did not show much variation during the entire period of bioassays. The mean dissolved oxygen content during the toxicity tests of dimethoate varied from 3.98 to 4.56 mg/L. The main factor due to which the physicochemical parameters did not vary during bioassay could be the atmospheric temperature conditions and nonavailability of feed to fishes during experiments, since the experiments were set in winter which did not lead to much variations in oxygen and carbon dioxide levels. Moreover, feeding stopped prior to the experiment prevented the deterioration of water quality. Therefore, it can be suggested that the hypoxic conditions did not prevail in the aquarium water during entire bioassay and mortality of fish was solely due to the pesticides intoxication. Our findings differ from the study conducted by [20] that reported significant decrease in oxygen consumption between control and treated groups of* Puntius chola* exposed to chlorpyrifos. They reported increase in stress conditions due to oxygen consumption during 96-hour static bioassay. However, the excessive secretion of mucous decreases the gaseous exchange through gills which reduces the ability of fish to utilize dissolved oxygen from the water. The increased utilization of oxygen and reduced supply of it may cause a hypoxic condition in fish [22, 27]. Furthermore, increase in total dissolved solids on 2nd and 3rd days could be attributed to the fact that the feed left in the fish body might have been secreted which resulted in its increase. On the 3rd day, all the feed would be secreted out of the fish body which restricted its further increase in the aquarium. The increase in total dissolved solids could also be due to the pesticide concentration in the aquarium.

The primary target of organophosphates (OPs) is the neuroinhibitory nature of acetylcholinesterase (AChE) enzyme in the synaptic cleft which leads to paralysis in acute toxicity exposures. However, the OP toxicity in natural waterbodies where fishes are exposed chronically to these xenobiotics induces irreversible long term effects leading to physiological dysfunctions in them. Like other organophosphates, dimethoate inhibits acetylcholinesterase (AChE) which is present in mammals, fish, birds, and insects. AChE is a class of enzymes which initiate the hydrolysis of acetylcholine (ACh), a neurotransmitter, into inactive choline and acetic acid [28]. The inhibition creates a build-up of acetylcholine at the nerve synapses disabling the enzyme cholinesterase which is vital for a functioning central nervous system [29]. The continuous accumulation of acetylcholine leads to loss of balance, convulsions, paralytic symptoms, and eventually death. During present investigation, the slowing down of motion, uncoordinated movements, and convulsion in the initial phase of bioassay may be attributed to the accumulation of acetylcholine in nerve synapses which grew more with the advent of exposure time of the pesticide and ultimately collapse to the bottom of the aquarium showing the signs of paralysis. The excess secretion of mucus over the body could be the first-line defence mechanism reactions elicited by host towards the stress conditions. Our findings are in agreement with the findings of [18] that reported abnormal behavioural changes such as restlessness, aggregations at one corner of the aquarium, erratic and jerky swimming, frequent surfacing, increased mucous secretions, and loss of balance in* Danio rerio* exposed to dimethoate. Increase in the respiratory movements was noticed during entire period of bioassay, while fishes became inactive and almost nonmotile with clinical signs of fading of body colour, erosion of scales, lesions, and hemorrhagic patches all over the body especially on the ventral side. Similar changes were noticed by [23] that reported increased opercular movement, sluggish, lethargic, and abnormal swimming, loss of buoyancy, muscular tetany, and fading of body colour in* Heteropneustes fossilis* exposed to dimethoate. Increase in opercular movement may be to overcome the hypoxic conditions which arise due to damage of gills by the pesticide which hampers oxygen uptake [30]. Exposure studies (concentrations from 2.5 to 4.0 mg/L) using catfish (*Heteropneustes fossilis*) observed altered swimming behaviour, increased gulping for air, and increased mucus secretion over the body. In addition, the fishes were highly sensitive to low concentrations with a 96 h LC_50_ of 2.98 mg/L [31]. Bruise in the caudal fin of fish and on ventral side was occasionally seen at the time close to the termination of the experiments. It is due to the fact that ventral side of the body has fewer scales as compared to dorsal side which facilitates easy absorption of the pesticide dermally. Steep slope functions of toxicity curves of 96 h mortality concentration data for dimethoate indicate a large increase in the mortality associated with the relatively small increase in the concentration of this pesticide. This may be due to the rapid absorption of the pesticide and rapid onset of effects [32]. The erosion of scales, dark or pale colouration of the body, and hemorrhagic patches all over the body could be due to the dermal absorption of the pesticide after long exposure (Table 3).

## 6. Conclusion

Results obtained in the present study suggest that the values of median lethal concentrations of dimethoate are alarming with wide range of toxicity among fish. Therefore, entry of pesticides from point and nonpoint sources of pollution into our natural waterbodies must be checked so as to avoid the contamination in our natural ecosystems.

## Figures and Tables

**Table 1:** Cumulative LC_50_ values of range finding (RF) tests of dimethoate.

S. number	RF test of dimethoate	LC_50 _values (ppm)	95% confidence limits		Regression equation *Y* = *a* − *bX*	Coefficient of determination *R* ^2^
			Upper fiducial limit	Lower fiducial limits		
(1)	*r*_1_	1.08	2.01	0.92	*Y* = 0.921 − 0.020*X*	0.95
(2)	*r*_2_	1.16	1.98	1.02	*Y* = 1.01 − 0.042*X*	0.9412
(3)	*r*_3_	1.06	2.01	0.96	*Y* = 1.62 − 0.072*X*	0.9259

		Mean = 1.1 ppm				

**Table 2:** Means and SD of water quality parameters of aquarium water during dimethoate bioassay on different intervals.

S. number	Water quality parameter	Day			
		1	2	3	4
(1)	Lab. temperature (°C)	13 ± 0.0	12 ± 0.0	13 ± 0.0	13 ± 0.0
(2)	Water temperature (°C)	11 ± 0.0	11 ± 0.0	12 ± 0.0	11 ± 0.0
(3)	Dissolved oxygen (mg/L)	4.56 ± 0.55	4.0 ± 0.10	3.98 ± 0.19	3.9 ± 0.13
(4)	CO_2_ (mg/L)	2 ± 0.0	2 ± 0.0	2 ± 0.0	2 ± 0.0
(5)	pH	6.98 ± 0.26	6.95 ± 0.13	7.05 ± 0.26	6.9 ± 0.12
(6)	Total dissolved solids (mg/L) × 10^3^	2.83 ± 0.81	2.66 ± 1.03	3 ± 1.09	2.66 ± 1.4

**Table 3:** Behavioural responses elicited by common carp during bioassay.

Time	Response						
	Uncoordinated movements	Convulsions	Mucus secretion	Imbalanced swimming	Erosion of scales	Body colour	hemorrhagic patches
6 hours	+****+	−	−	+	−	−	−
12 hours	+****+	+	−	+	−	−	−
24 hours	+**** + ****+	+****+	+	+**** + ****+	−	−	−
48 hours	−	−	+	+**** + ****+	+	−	+****+
72 hours	−	−	+****+	+**** + ****+	+****+	+****+	+****+
96 hours	−	−	+**** + ****+	+**** + ****+	+**** + ****+	+**** + ****+	+**** + **** + ****+

+, feeble; ++, progressive; +++, intense; ++++, severe.

## References

[1] Bhat A. R., Wani M. A., Kirmani A. R. (2010). Brain cancer and pesticide relationship in orchard farmers of Kashmir. *Indian Journal of Occupational and Environmental Medicine*.

[2] Baba S. H., Wani M. H., Zargar B. A., Wani S. A., Kubrevi S. S. (2012). Pesticide delivery system in apple growing belt of Kashmir valley. *Agricultural Economics Research Review*.

[3] Alkahem H. F., Ahmed Z., Al-Akel A. S., Shamsi M. J. K. (1998). Toxicity bioassay and changes in haematological parameters of *Oreochromis niloticus* induced by trichlorfon. *Arab Gulf Journal of Scientific Research*.

[4] Rahman M. Z., Hossain Z. M. F. (2002). Effect of diazinon 60 EC on *Anabas testudineus, Channa punctatus* and *Barbodes gonionotus*. *NAGA, The ICLARM Quarterly*.

[5] Ansari S., Ansari B. A. (2011). Embryo and fingerling toxicity of dimethoate and effect on fecundity, viability, hatchability and survival of zebrafish, *Danio rerio* (Cyprinidae). *World Journal of Fish and Marine Sciences*.

[6] Velmurugan B., Selvanayagam M., Cengiz E. I., Unlu E. (2007). The effects of monocrotophos to different tissues of freshwater fish *Cirrhinus mrigala*. *Bulletin of Environmental Contamination and Toxicology*.

[7] Sailaja V., Suneetha T., Ramanamma T. (2015). Effect of pesticides on edible aquatic organisms. *Advance Research Journal of Biological Sciences and Molecular Techniques*.

[8] Anees M. A. (1978). Haematological abnormalities in a freshwater teleost, *Channa punctatus* (Bloch), exposed to sub-lethal and chronic levels of three organophosphorous insecticides. *International Journal of Ecology and Environmental Sciences*.

[9] Campagna A. F., Eler M. N., Espíndola E. L. G., Senhorini J. A., Do Rêgo R. F., Silva L. O. L. (2006). Dimethoate 40% organosphosphorous pesticide toxicity in *Prochilodus lineatus* (Prochilodontidae, Characiformes) eggs and larvae. *Brazilian Journal of Biology*.

[10] Lundebye A.-K., Curtis T. M., Braven J., Depledge M. H. (1997). Effects of the organophosphorous pesticide, dimethoate, on cardiac and acetylcholinesterase (AChE) activity in the shore crab *Carcinus maenas*. *Aquatic Toxicology*.

[11] Ganeshwade R. M. (2011). Biochemical changes induced by dimethoate in the liver of fresh water fish puntius ticto (HAM). *Biological Forum ó*.

[12] Adoni A. D., Joshi G., Ghosh K. (1985). *Workbook on Limnology*.

[13] Banaee M., Mirvaghefi A. R., Ahmadi K., Banaee S. (2008). Determination of LC50 and investigation of acute toxicity effects of diazinon on hematology and serology indices of common carp (*Cyprinus carpio*). *Journal of Marine Science and Technology Research*.

[14] Pandey N. B., Chanchal A. K., Singh S. B., Prasad S., Singh M. P. Effect of some biocide on blood and oxygen consumption of *C. punctatus* (Bloch).

[15] Oruç E. Ö., Usta D. (2007). Evaluation of oxidative stress responses and neurotoxicity potential of diazinon in different tissues of *Cyprinus carpio*. *Environmental Toxicology and Pharmacology*.

[16] Ganeshwade R. M. (2012). Biochemical changes induced by dimethoate (Rogor 30% EC) in the gills of fresh water fish *Puntius ticto* (Hamilton). *Journal of Recent Trends in Bioscience*.

[17] Qayoom I., Balkhi M. H., Mukhtar M., Bhat F. A., Shah F. A. (2014). Biochemical toxicity of organophosphate compounds in fishes. *SKUAST Journal of Research*.

[18] Üner N., Oruç E. Ö., Sevgiler Y., Şahin N., Durmaz H., Usta D. (2006). Effects of diazinon on acetylcholinesterase activity and lipid peroxidation in the brain of *Oreochromis niloticus*. *Environmental Toxicology and Pharmacology*.

[19] Menzie C. M. (1969). *Metabolism of Pesticides. U. S. Department of the Interior, Bureau of Sport Fisheries and Wildlife*.

[20] Rand G. M., Petrocelli S. R. (1985). *Fundamentals of Aquatic Toxicology, Methods and Applications*.

[21] Svoboda M., Lusková V., Drastichová J., Žlábek V. (2001). The effect of diazinon on haematological indices of common carp (*Cyprinus carpio* L.). *Acta Veterinaria Brno*.

[22] Begum G., Vijayaraghavan S. (1995). *In vivo* toxicity of dimethoate on protein and transaminases in the liver tissue of fresh water fish *Clarias batrachus* (Linn). *Bulletin of Environmental Contamination and Toxicology*.

[23] Fukuto T. R. (1990). Mechanism of action of organophosphorus and carbamate insecticides. *Environmental Health Perspectives*.

[24] Qayoom I. (2015). *Residue Analysis of Pesticides in Dal waters and their effect on common carp fish, Cyprinus carpio var. communis [Ph.D. thesis]*.

[25] Reish D. J., Oshida O. S. (1987). *Manual of Methods in Aquatic Environment Research. Part 10, Short Term Bioassay*.

[26] APHA (2005). *Standard Methods for the Examination of Water and Wastewater*.

[27] Adedeji O. B., Okocha R. O. (2012). Overview of pesticide toxicity in fish. *Advances in Environmental Biology*.

[28] Hoegberg E. I., Cassaday J. T. (1951). The reaction of O,O-dialkyl thiophosphoric acid salts with some *α*-haloacyl derivatives. *Journal of the American Chemical Society*.

[29] Verma S. R., Pal N., Tyagi A. K., Dalela R. C. (1979). Toxicity of swascol® 1P (SLS) to *Channa punctatus* and *Cirrhina mrigala*: biochemical alterations. *Bulletin of Environmental Contamination and Toxicology*.

[30] Pandey R. K., Singh R. N., Singh S., Singh N. N., Das V. K. (2009). Acute toxicity bioassay of dimethoate on freshwater airbreathing catfish, *Heteropneustes fossilis* (Bloch). *Journal of Environmental Biology*.

[31] Srivastava A. K., Mishra D., Shrivastava S., Srivastav S. K., Srivastav A. K. (2010). Acute toxicity and behavioural responses of *Heteropneustes fossilis* to an organophosphate insecticide, dimethoate. *International Journal of Pharma and Bio Sciences*.

[32] Verma V. K., Saxena A. (2013). Investigations on the acute toxicity and behavioural alterations induced by the organophosphate pesticide, chlorpyrifos on *Puntius chola* (Hamilton-Buchanan). *Indian Journal of Fisheries*.

